# A Genome-Wide Screen for Bacterial Envelope Biogenesis Mutants Identifies a Novel Factor Involved in Cell Wall Precursor Metabolism

**DOI:** 10.1371/journal.pgen.1004056

**Published:** 2014-01-02

**Authors:** Catherine Paradis-Bleau, George Kritikos, Katya Orlova, Athanasios Typas, Thomas G. Bernhardt

**Affiliations:** 1 Department of Microbiology, Infectiology and Immunology, Université de Montréal, Montréal, Québec, Canada; 2 European Molecular Biology Laboratory, Genome Biology Unit, Heidelberg, Germany; 3 Department of Microbiology and Immunology, University of California at San Francisco, San Francisco, California, United States of America; 4 Department of Microbiology and Immunobiology, Harvard Medical School, Boston, Massachusetts, United States of America; Indiana University, United States of America

## Abstract

The cell envelope of Gram-negative bacteria is a formidable barrier that is difficult for antimicrobial drugs to penetrate. Thus, the list of treatments effective against these organisms is small and with the rise of new resistance mechanisms is shrinking rapidly. New therapies to treat Gram-negative bacterial infections are therefore sorely needed. This goal will be greatly aided by a detailed mechanistic understanding of envelope assembly. Although excellent progress in the identification of essential envelope biogenesis systems has been made in recent years, many aspects of the process remain to be elucidated. We therefore developed a simple, quantitative, and high-throughput assay for mutants with envelope biogenesis defects and used it to screen an ordered single-gene deletion library of *Escherichia coli*. The screen was robust and correctly identified numerous mutants known to be involved in envelope assembly. Importantly, the screen also implicated 102 genes of unknown function as encoding factors that likely impact envelope biogenesis. As a proof of principle, one of these factors, ElyC (YcbC), was characterized further and shown to play a critical role in the metabolism of the essential lipid carrier used for the biogenesis of cell wall and other bacterial surface polysaccharides. Further analysis of the function of ElyC and other hits identified in our screen is likely to uncover a wealth of new information about the biogenesis of the Gram-negative envelope and the vulnerabilities in the system suitable for drug targeting. Moreover, the screening assay described here should be readily adaptable to other organisms to study the biogenesis of different envelope architectures.

## Author Summary

Bacteria are surrounded by complex structures called cell envelopes that play an essential role in maintaining cellular integrity. Organisms classified as Gram-negative have especially complicated envelopes that consist of two membranes with a tough cell wall exoskeleton sandwiched between them. This envelope architecture is extremely proficient at preventing drug molecules from entering the cell. Gram-negative bacteria are therefore intrinsically resistant to many antibiotics, limiting the therapeutic options for treating infections caused by these organisms. To reveal new weaknesses in the Gram-negative envelope for drug targeting, we developed a quantitative, high-throughput assay for mutants with envelope biogenesis defects and used it to screen an ordered single-gene deletion library of the model Gram-negative bacterium *Escherichia coli*. Importantly, the screen implicated 102 genes of previously unknown function as encoding factors that likely participate in envelope biogenesis. As a proof of principle, one of these factors, ElyC (YcbC), was characterized further and shown to play a critical role in the metabolism of the essential lipid carrier used for cell wall synthesis. Further study of ElyC function and that of other factors identified in our screen is likely to reveal novel ways to disrupt the envelope assembly process for therapeutic purposes.

## Introduction

The cell envelope of bacteria serves as their interface with the environment. This structure plays an essential role in maintaining cellular integrity and provide protection from external insults. For pathogens, the envelope is the site of first contact with the host and where major pathogenicity determinants such as adhesins and toxin secretion systems assemble [1]–[3]. Uniquely bacterial in origin, cell envelope building blocks are also recognized by host innate immune receptors as signs of invasion [4]. The cell envelope is therefore both a strength and a weakness for pathogenic organisms. Similarly, these structures simultaneously present great challenges and opportunities for therapeutic intervention. Many otherwise effective drugs have difficulty penetrating bacterial envelopes to reach their cellular target [5]. On the other hand, molecules like penicillin and vancomycin that disrupt envelope assembly processes have been some of our most effective antibacterial treatments.

Although envelope composition varies throughout the bacterial domain, the structures are typically complex and multi-layered [6], [7]. The two most well-studied classes of envelopes belong to the *Firmicutes* and *Proteobacteria* and are traditionally referred to as being either Gram-positive or Gram-negative, respectively, based on how they normally react to the classic Gram-staining procedure. Gram-positive (monoderm) envelopes are bi-layered structures consisting of a single membrane surrounded by a thick cell wall composed of peptidoglycan (PG) and teichoic acids [7]. The envelopes of Gram-negative bacteria (diderm), on the other hand, have three layers: an inner (cytoplasmic) membrane, an outer membrane, and a thin layer of PG sandwiched between them [7]. *Mycobacteria* possess another distinct envelope class. In addition to a cell membrane and PG layer, they contain a second polysaccharide layer called the arabinogalactan, which is attached to waxy hydrocarbons called mycolic acids that are thought to form the equivalent of the Gram-negative outer membrane [8].

The outer membrane of Gram-negative proteobacteria provides these organisms with a high intrinsic resistance to antibiotics [9]. Thus, therapeutic options for treating Gram-negative bacterial infections are relatively limited. The problem has worsened significantly in recent years with the emergence of carbapenem-resistant Gram-negative *Enterobacteriaceae* like *Klebsiella pneumoniae* and *Escherichia coli* [10]. It is therefore important that new vulnerabilities in the Gram-negative envelope be identified to serve as targets for antibacterial drugs, or for the development of inhibitors that disrupt the permeability barrier to sensitize resistant organisms to approved therapeutics. Along these lines, tremendous progress has been made in our understanding of Gram-negative envelope assembly over the last two decades [11]. Most of the essential envelope biogenesis systems have now been identified in *E. coli* and related proteobacterial pathogens including: (i) the Sec system that transports proteins across the inner membrane or inserts them into it, (ii) the Lol system for lipoprotein transport to the outer membrane, (iii) the Bam system for outer membrane beta-barrel protein assembly, (iv) the Lpt system for lipopolysaccharide (LPS) transport to and assembly in the outer membrane, and (v) the penicillin-binding proteins (PBPs) and associated factors that construct the PG layer [7]. What remains unclear is how these different processes are controlled and coordinated with one another so that the envelope grows uniformly and maintains its integrity as it is remodeled. Given that genes coding for envelope proteins constitute roughly one quarter of the *E. coli* genome, and that over a third of these have an unknown or poorly understood function [12], it is likely that many factors important for modulating envelope assembly remain to be identified. Large-scale genetic methods represent a promising avenue for discovering these factors.

**Figure 1:**
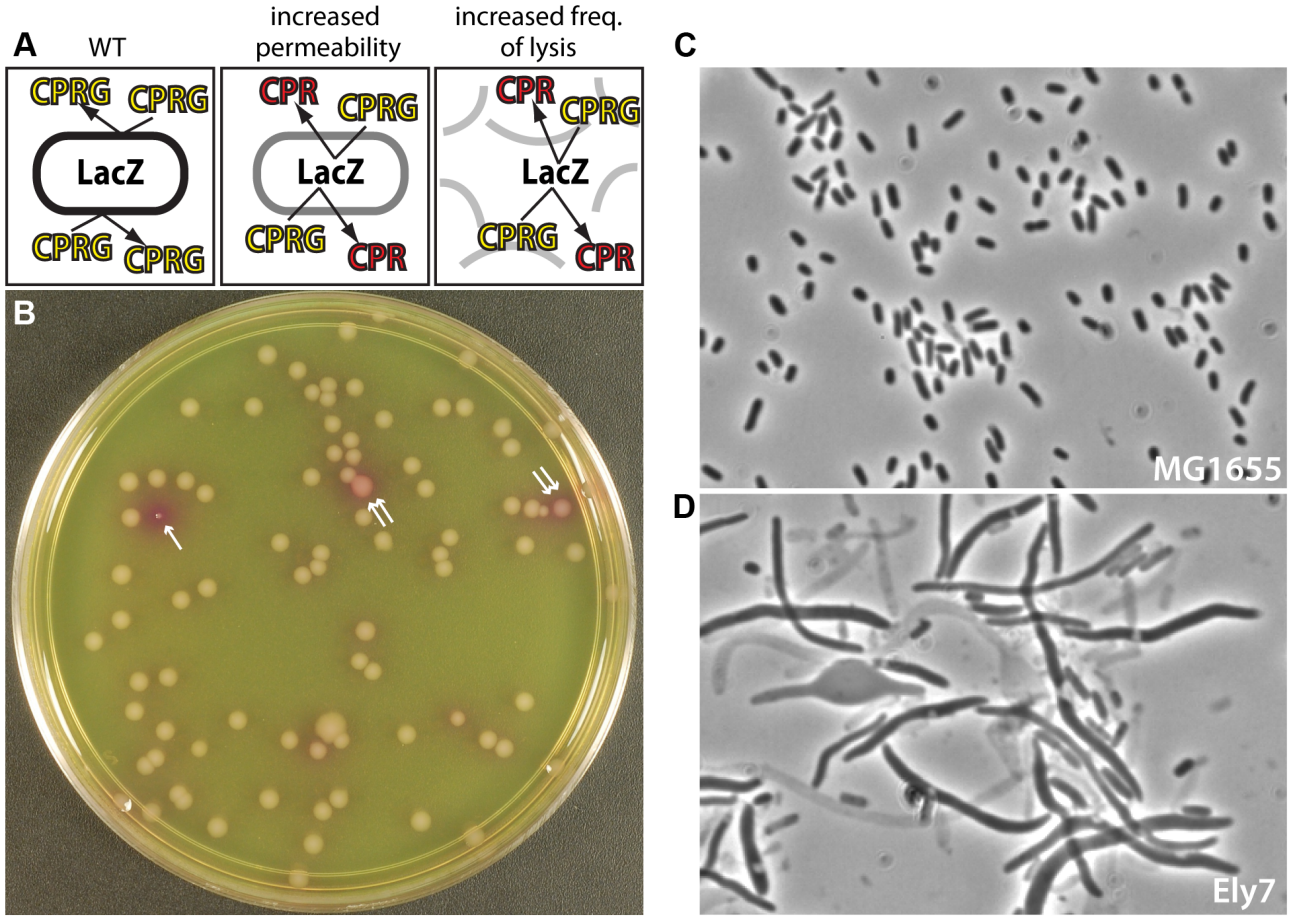
A screen for mutants defective in cell envelope assembly. A. Shown is a schematic illustrating the logic of the screen. Wild-type (WT) cells are unable to cleave CPRG because the enzyme (LacZ) is separated from its substrate (CPRG) by the intact cell envelope and thus remain white. Mutants that lyse or have defects in envelope permeability are able to cleave CPRG and turn red. B. Picture of CPRG agar with colonies from an *E. coli* transposon mutant library. Most colonies are “white”, with occasional red (single arrow) or pink (double arrow) colonies identifying mutants likely to have envelope defects. The particular “red” mutant shown also has a growth defect. The plate was incubated overnight at 30°C. Note that we were unable to carry out the screen at 37°C due to excessive background color development on the CPRG agar. C–D. Micrographs show cells from a colony of WT (MG1655) (C) or an Ely mutant (Ely7) (D) grown on LB agar prepared with 1% NaCl. The transposon in Ely7 was mapped to the *nhaA* gene coding for a sodium-proton antiporter. The phenotype shown was observed on LB with 1% NaCl, but not on LB lacking added salt (data not shown). Cells were imaged on 1.2% agarose pads with a Nikon 50i microscope with a 100× phase-contrast objective.

Genetic screens for envelope biogenesis mutants were performed many years ago taking advantage of the release of periplasmic RNase from defective cells, which was detected as a zone of clearing on RNA-containing agar plates [13], [14]. However, these “periplasmic leaky” screens were performed in the pre-genomic era and only identified a small handful of mutants, some of which were never precisely mapped [15]–[18]. We therefore thought that revisiting this genetic approach with our current knowledge and technology would be fruitful for the identification of new factors involved in the biogenesis of the Gram-negative envelope. Rather than rely on the detection of RNase leakage, which requires replica-plating and the use of RNA-containing soft agar overlays [13], [14], we decided to use an old reporter, β-galactosidase (LacZ), in a new way. The β-galactosidase substrate chlorophenyl red-β-D-galactopyranoside (CPRG) fails to penetrate the *E. coli* envelope and cannot be processed by cytoplasmic LacZ (Figure 1). Because mutants impaired for envelope biogenesis typically either lyse at an elevated frequency to release LacZ into the medium and/or are more permeable to small hydrophobic molecules, we reasoned that they should be readily identifiable based on CPRG hydrolysis and the formation of red colonies on CPRG-containing agar. A preliminary screen of a transposon-mutagenized wild-type *E. coli* strain proved this to indeed be the case. We then proceeded to systematically screen an ordered *E. coli* deletion library for mutants with a CPRG+ phenotype, implicating numerous new factors in proper envelope assembly. As a proof of principle, we further analyzed a mutant inactivated for *ycbC*, which encodes a factor with a highly conserved domain of unknown function (DUF218) [19]. Loss of YcbC function was found to cause a severe growth defect at low temperature accompanied by an elevated frequency of cell lysis resulting from impaired metabolism of the essential lipid precursor required for PG biogenesis. We have therefore renamed this factor ElyC (elevated frequency of lysis) to reflect this phenotype. Further analysis of the function of ElyC and other CPRG+ mutants is likely to uncover a wealth of new information about the biogenesis of the Gram-negative envelope and its control under different environmental conditions. Importantly, the CPRG screen described here should also be transferable to other organisms to study the biogenesis of different envelope architectures and understand how one of the most rapidly evolving features of the bacterial cell adapts to different niches.

## Results

### A high-throughput screen for mutants defective in envelope biogenesis

Our goal was to develop a simple screen for the identification of new factors required for Gram-negative cell envelope biogenesis. We thought that β-galactosidase (LacZ) would be a useful reporter for envelope integrity because the classic protocol for measuring LacZ activity with o-nitrophenyl-β-D-galactopyranoside (ONPG) requires a membrane permeabilization step to allow substrate entry in cells [20]. We therefore reasoned that in the absence of membrane permeabilization, mutants impaired in envelope biogenesis could be identified by their enhanced LacZ activity over normal cells. The synthetic substrate CPRG was chosen over ONPG for screen development because of its increased sensitivity [21] and the red color of its cleavage product, CPR (chlorophenyl red), which we assumed would be easier to detect on LB agar than the yellow ONPG product. Although it is unclear which membrane of the Gram-negative envelope is primarily responsible for preventing CPRG from entering cells, the outer membrane is a good candidate given the hydrophobic nature of the synthetic substrate and the well-known effectiveness of this layer at blocking uptake of other hydrophobic molecules. Thus, mutants capable of processing CPRG may either be defective in the permeability barrier of the outer membrane or possess a defect that results in an elevated frequency of cell lysis promoting the release of LacZ into the medium (Figure 1A).

As an initial test of the screen, we mutagenized wild-type *E. coli* MG1655, which is Lac^+^, with the EzTn-Kan transposome (Epicentre) and plated dilutions of the resulting mutant library on LB agar supplemented with CPRG (20 µg/ml) and IPTG (50 µM) to induce the *lac* operon. Following overnight incubation at 30°C, mutant colonies ranging from pink to dark red with intense halos were observed at a frequency of approximately 1–2% (Figure 1B, and data not shown). These CPRG+ colonies were purified on LB agar prepared with a range of NaCl concentrations (0, 0.5, and 1%) to assess growth of the mutants under different osmotic conditions. Cells from the resulting colonies were visualized by phase contrast microscopy, and mutant strains displaying an elevated frequency of lysis were given the designation Ely. Figure 1D shows an example micrograph of an isolate with a severe Ely phenotype relative to wild-type (Figure 1C). Several mutants, including the example shown in Figure 1D, displayed lysis and/or morphological phenotypes that varied greatly in their magnitude depending on the NaCl concentration of the medium or the growth temperature, thus highlighting the utility of testing the different growth conditions (data not shown).

Transposon insertion sites were mapped in approximately 100 CPRG+ strains, many of which displayed some level of cell lysis in micrographs. A large fraction of the mutants harbored insertions in genes known to encode envelope assembly factors, including: (i) components of the Tol-Pal system [22], [23], (ii) the Tat transport system [24], [25], (iii) PBP1b and its partner LpoB [26], [27], and (iv) EnvC [28]–[30]. Thus, the screen was identifying factors important for envelope assembly as intended. One problem with the transposon mutagenesis approach, however, was that insertions in large genes or operons represented a large fraction of the CPRG+ isolates. For example, over 10% of the isolates had insertions in the *tol-pal* locus (data not shown). This observation suggested that screening an ordered mutant collection would likely result in a more comprehensive identification of envelope defective mutants than random mutagenesis and screening. We therefore set out to screen a defined mutant library [31] that includes the *E. coli* knockout (Keio) collection [32] as well as a collection of mutants with hypomorphic alleles of essential genes and mutants lacking genes for small RNAs.

The parent strain background (BW25113) of the ordered mutant library is LacZ^−^. We thus could not directly use the ordered library for CPRG screening. To convert the library to LacZ^+^ *en masse*, we constructed a mobile plasmid, pCB112, encoding *lacZ* under control of the lactose promoter (P_lac_). Lawns of a pCB112-containing donor strain were prepared, and the defined mutant collection was then transferred onto the lawns in 384-pin format. After overnight incubation, spots corresponding to the locations of pinned library cells were transferred to agar supplemented with kanamycin (Kan) and chloramphenicol (Cam) to select for exconjugants possessing both the defined mutation (marked by a Kan^R^ cassette) and the *lacZ* plasmid (marked by a Cam^R^ cassette). Doubly drug-resistant colonies were then transferred in 384-pin format to LB agar supplemented with IPTG and CPRG to screen for envelope mutants. Based on our experience with the transposon library screen, we decided to screen the ordered mutant set at room temperature and 30°C on media with different NaCl concentrations (0 and 1% NaCl for both temperatures) in order to maximize the number of identified envelope biogenesis factors and potentially identify factors required for adaptation to different temperatures or osmotic conditions.

**Figure 2:**
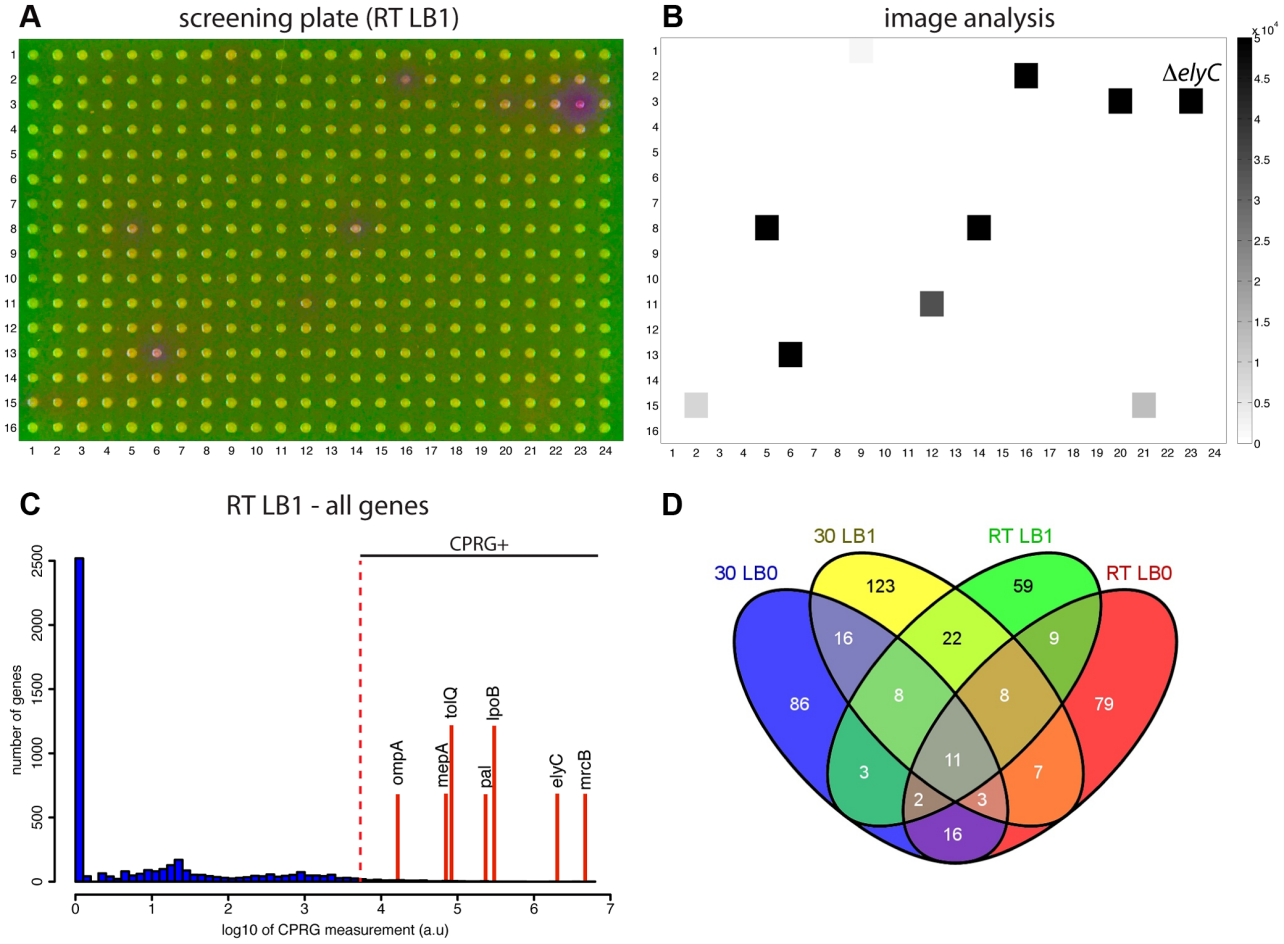
Using the CPRG assay in high-throughput. A. Picture of indicator agar (1% NaCl) with pin-transferred cells of the ordered library converted to Lac^+^ by conjugation. Plate was incubated for 23 hrs at room temperature. B. Output of the image analysis software (Iris) for the plate shown in (A). C. CPRG assay score distribution for the screen carried out at room temperature on agar prepared with 1% NaCl. Positions of genes of interest and/or known importance for envelope integrity are indicated by the red lines. Genes with scores above the cut-off (10^3.7^ units) were designated as CPRG+ hits. D. Venn diagram comparing the hits identified in the different growth conditions.

To identify potential envelope biogenesis mutants, plate images from each growth condition were quantitatively analyzed for red color development using a custom software application called Iris that can quantify several different colony features including color (Kritikos & Typas, unpublished data). The observed CPR color development on the screening plates was found to be directly proportional to incubation time (data not shown). However, because the color diffuses, an early time-point was used for quantification to avoid neighbors of CPRG+ colonies from being scored as hits. An example of a screening plate and corresponding quantification is shown in Figure 2A–B. These panels as well as the CPRG score distributions for all 4 different conditions (Figure 2C and S1) clearly show that the assay is specific (only a small proportion of mutants score positively) and has a wide dynamic range. Following quantification of all plates and setting the CPRG score threshold at 10^3.7^ units, 120–200 CPRG+ mutants were identified in each condition (Table S1), with the majority (∼75%) being condition-specific (Figure 2D). For example, mutants with defects in enterobacterial common antigen (ECA) biogenesis were detected as CPRG+ on LB no NaCl, while mutants defective for outer membrane biogenesis, the Tol-Pal machinery, and colanic acid biosynthesis were scored as positive on LB with 1% NaCl. On the other hand, LPS biosynthesis genes were CPRG+ at low temperature. Surprisingly, mutants involved in homologous recombination were also enriched in LB containing salt. The reason for this is not clear at present. Functional enrichment analysis of Gene Ontology (GO) and KEGG pathways indicated that the terms envelope, membrane, cell-wall, peptidoglycan, and cell surface structure-related were the most statistically significant terms associated with hits from all four datasets (Table S2 and S3). Although many factors involved in outer membrane biogenesis were identified in the screen (Table S1 and S2), it remains unclear whether they were identified due to the loss of the outer membrane barrier function or because mutants defective for these factors lyse at an elevated frequency. In any case, the screen was clearly effective at identifying factors involved in many different aspects of envelope biogenesis/assembly. Importantly, 102 CPRG+ mutants (∼22% of all hits) were in genes coding for proteins of unknown function (Table S4). For more than half of these factors, no significant growth phenotype was observed when the same library was subjected to over 300 different plating conditions [31]. This suggests that the CPRG readout could be a very sensitive and powerful assay to use in larger-scale phenotyping screens for discovering gene function and network architecture in a guilt-by-association manner [31].

### Loss of ElyC function results in cell lysis at low temperature

**Figure 3:**
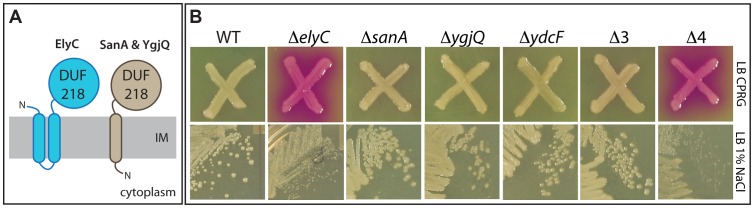
Phenotypes of mutants inactivated for DUF218 factors. A. Schematic showing the predicted membrane topologies of ElyC and its paralogues SanA and YgjQ. The fourth paralogue, YdcF, is predicted to be cytoplasmic and is not shown. Topology predictions were performed using the TMHMM server (http://www.cbs.dtu.dk/services/TMHMM/). B. CPRG and growth phenotypes of deletion mutants lacking DUF218 factors. Cells of the indicated deletion mutants in an MG1655 strain background were either patched onto CPRG indicator agar (20 µg/ml CPRG and 50 µM IPTG, upper panels) or streaked onto LB agar prepared with 1% NaCl (lower panels). All plates were incubated at room temperature.

One of the mutants with the most striking CPRG+ phenotype was a deletion of the gene of unknown function *ycbC*. Its phenotype was strongest at room temperature, and based on the elevated frequency of lysis observed in cell populations from colonies (not shown), we renamed the gene *elyC*. The *elyC* reading frame encodes a protein with two predicted transmembrane domains and a large domain of unknown function (DUF) designated as a DUF218 domain in the Pfam database [19]. Topology predictions indicate that the DUF218 domain of ElyC is likely to be periplasmic (Figure 3A). Interestingly, DUF218 domains are abundant and widely distributed in the bacterial domain [19], but little is known about their biological activity. The CPRG screen indicated a potentially prominent role for ElyC in envelope assembly, so as a proof of principle we initiated a more detailed analysis of its function as well as the function of its paralogues.

*E. coli* encodes four proteins annotated as possessing a DUF218 domain: ElyC, SanA, YgjQ, and YdcF [19]. All of these factors are predicted to be integral membrane proteins with periplasmic domains (Figure 3A) with the exception of YdcF, which is predicted to be cytoplasmic. SanA was originally identified because its overproduction suppressed the vancomycin sensitivity of an uncharacterized *E. coli* mutant with an envelope permeability defect [33]. The protein was also found to play a role in vancomycin resistance of wild-type cells at high temperature (43°C and above), and the inactivation of its orthologue SfiX in *Salmonella typhimurium* was found to suppress the cell division defect induced by HisHF overproduction (the His^C^ pleiotropic response) [33], [34]. These observations suggested a potential yet undefined role for SanA in envelope biogenesis [33], [34]. The crystal structure of the related YdcF protein was reported several years ago, revealing a fold for the DUF218 domain resembling that of the adenine nucleotide alpha hydrolase-like family [35]. It was also reported that YdcF binds S-adenosyl-L-methionine, but the physiological relevance of this observation is not clear [35].

To further investigate the effect of inactivating DUF218 factors, deletion mutations from the Keio collection were transduced to a wild-type (MG1655) strain background and their growth and CPRG phenotypes were assessed. As expected from the screening results, the only single mutation that resulted in a CPRG+ phenotype on indicator agar was Δ*elyC* (Figure 3B). It was also the only single mutation that caused an observable growth phenotype. Compared to wild-type, the Δ*elyC* mutant grew normally at 30, 37 and 42°C, but grew poorly and formed very small colonies on LB agar plates incubated at room temperature (Figure 3B). The growth defect at this temperature was most severe on LB agar containing 1% NaCl, and became less pronounced at lower salt concentrations (data not shown). Importantly, the growth and CPRG+ phenotypes of the Δ*elyC* mutant were corrected by the expression of *elyC* in *trans* (see below), indicating that they were indeed the result of ElyC inactivation rather than an effect of the deletion on the expression of nearby genes. None of the other single mutants lacking a DUF218 factor displayed a growth or morphological phenotype on LB agar prepared with 0, 0.5, or 1% NaCl at any incubation temperature tested (room temperature, 30, 37, or 42°C) (data not shown). Moreover, a triple mutant (Δ3) lacking *sanA*, *ygjQ*, and *ydcF* also grew indistinguishably from wild-type under the conditions tested (Figure 3B). We also constructed a quadruple mutant (Δ4) lacking all DUF218 factors. The mutant was viable and displayed growth and CPRG phenotypes that were equivalent to the single *elyC* deletion (Figure 3B). Thus, inactivating additional DUF218 factors did not exacerbate the Δ*elyC* growth defect. Moreover, overproduction of other DUF218 factors did not correct the growth phenotypes resulting from ElyC inactivation, suggesting that the functions of the different proteins do not overlap (see below). We conclude that DUF218 factors are not required for viability and that, of the four such proteins produced by *E. coli*, only ElyC appears to play a significant role in envelope biogenesis as revealed by its low temperature growth and CPRG+ phenotypes.

**Figure 4:**
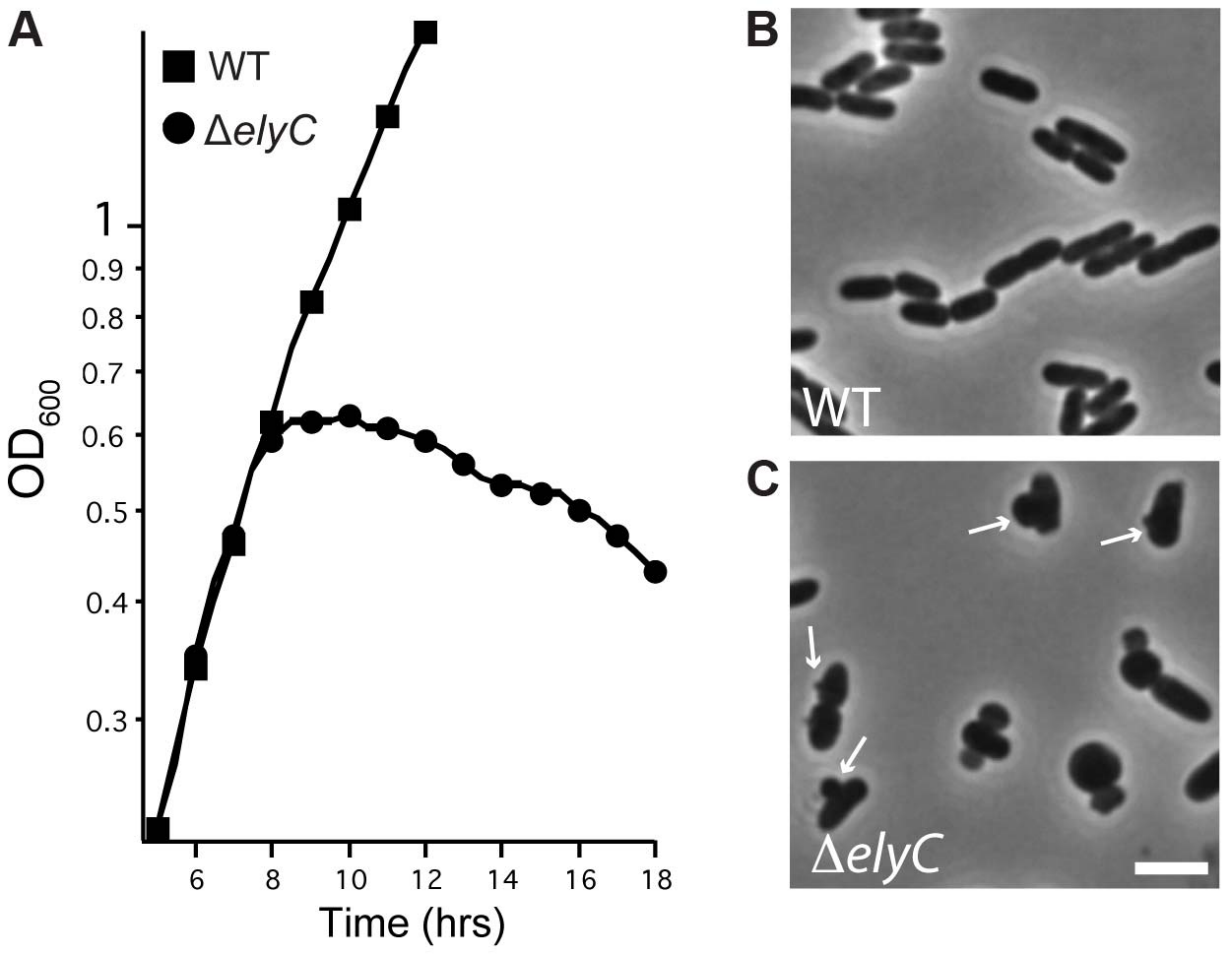
Loss of ElyC function results in lysis. A. Cells of MG1655 [WT] or CB152 [Δ*elyC*] were grown overnight in LB medium (1% NaCl) at 37°C, diluted 1∶100 and grown at 37°C to an OD_600_ of approximately 0.4. Cultures were then diluted to an OD_600_ of 0.04 in LB 1% NaCl and grown at room temperature in a shaking water bath. Cell growth was monitored by following culture OD_600_. Time is hours after the final inoculation. B–C. At approximately 8 hours post inoculation, cells from the indicated cultures were imaged on 1.2% agarose pads using a Nikon TE2000 microscope with a 100× phase contrast objective. Bar equals 3 microns.

To investigate the consequence of ElyC inactivation further, we monitored the growth and morphology of a Δ*elyC* mutant grown in liquid LB medium (1% NaCl) at room temperature. Interestingly, the mutant culture grew as rapidly as wild-type cells until it reached late-exponential phase when culture density abruptly stopped increasing and began a slow decline (Figure 4A). Microscopic analysis revealed that up until the point of growth divergence, the mutant cells had a morphology that was indistinguishable from wild-type cells (data not shown). However, visualization of Δ*elyC* cells harvested just after the decrease in culture density was observed revealed that the cells lysed via membrane blebs emanating from midcell or the cell quarter positions (Figure 4B and C). This morphological phenotype bears a striking resemblance to cells lysing following treatment with penicillin and other β-lactams [36]–[38], suggesting a potential role for ElyC in PG biogenesis.

### ElyC is required for proper cell wall biogenesis

**Figure 5:**
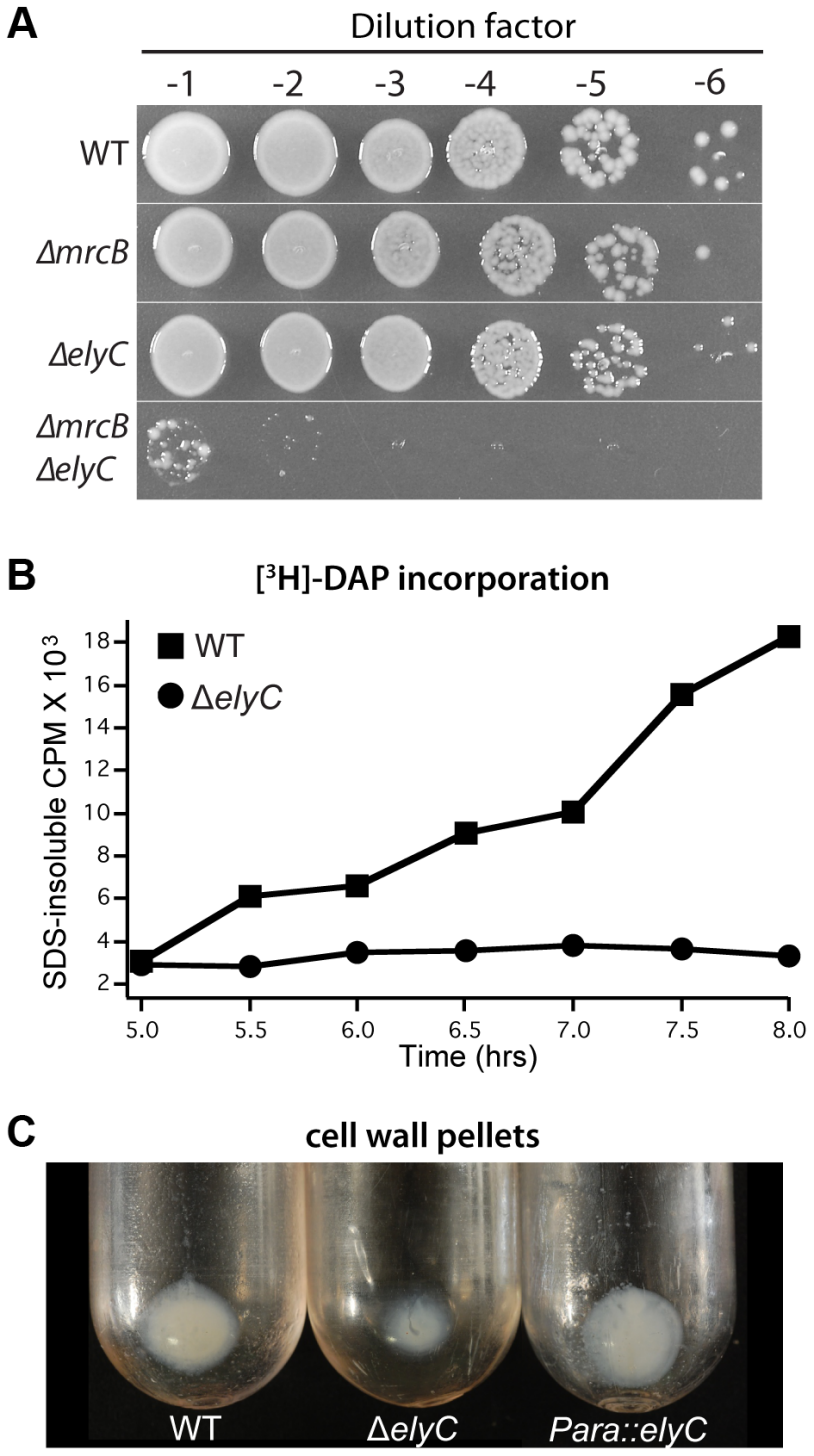
Mutants lacking ElyC have a PG synthesis defect. A. Overnight cultures of TB28 [WT], TU122 [Δ*mrcB*], CB152 [Δ*elyC*], and CB172 [Δ*elyC* Δ*mrcB*] were serially diluted following normalization for culture OD_600_. Five microliters of each dilution was spotted onto LB agar and plates were incubated for 3 days at room temperature. B. Cultures of CB74 [*lysA*::Tn*10*] and CB330 [Δ*elyC lysA*::Tn*10*] were grown as in Figure 4A in medium supplemented with [^3^H]-mDAP. PG synthesis was monitored by the incorporation of radioactivity into SDS-insoluble material. See text for details. C. Cultures (500 ml) of TB28 [WT], CB152 [Δ*elyC*], and CB152(attλCB118) [Δ*elyC* (P_ara_::*elyC*)] were grown at room temperature to an OD_600_ of 0.5 and cell wall pellets were prepared as described in Methods and Materials. Shown is a picture of the pellets following ultracentrifugation to sediment isolated sacculi. Similar preparations from cultures grown at 37°C showed no observable difference in pellet size (data not shown). Note that the initial pellet following SDS treatment of cells is likely to include material in addition to sacculi. Thus the magnitude of the reduction in pellet size is only a rough measure of the PG synthesis defect in the Δ*elyC* strain.

We reasoned that if ElyC is indeed important for PG assembly, its inactivation may be synthetically lethal with deletion mutants lacking PG synthases. Two important PG synthases produced by *E. coli* are the bifunctional (Class A) PBPs, PBP1a and PBP1b, encoded by the *mrcA* (*ponA*) and *mrcB* (*ponB*) genes, respectively [39]. These factors possess both peptidoglycan glycosyltransferase (PGT) activity to synthesize the glycan strands of PG and transpeptidase (TP) activity to crosslink the glycan chains via their attached peptide moieties [40]. Cells lacking either of these PBPs are viable, but the simultaneous inactivation of both factors results in rapid lysis and cell death [26], [27], [41], [42]. We were able to construct both Δ*elyC* Δ*mrcA* and Δ*elyC* Δ*mrcB* double mutants and maintain them at 37°C. However, consistent with a role for ElyC in PG biogenesis at lower temperatures, we found that Δ*elyC* was synthetically lethal with PBP1b inactivation but not in combination with Δ*mrcA* when the mutants were grown at room temperature (Figure 5A, data not shown).

To directly measure PG synthesis in a mutant lacking ElyC, we metabolically labeled cells with [^3^H]-*meso*-diaminopimelic acid (mDAP), an amino acid that is unique to the stem peptide of PG. The PG sacculus is one of the few cellular structures that remains insoluble in a boiling detergent solution (4% SDS). Therefore, upon [^3^H]-mDAP labeling, PG synthesis can be monitored by withdrawing aliquots of culture at times following label addition, boiling them in 4% SDS, and passing the solution through a 0.22 µm filter [43]. Radioactivity retained on the filter reflects the amount of [^3^H]-mDAP incorporated into the PG layer. For the labeling experiments, cultures of wild-type and ElyC^−^ cells were subcultured to an OD_600_ of 0.04 in LB 1% NaCl containing [^3^H]-mDAP and grown at room temperature. Incorporation of the radiolabel into SDS-insoluble material was then monitored over the time-course shown in Figure 5B. Strikingly, PG synthesis in ElyC^−^ cells appeared to be completely blocked relative to wild-type cells, with the first observable signs of inhibition detected at 5.5 hours of growth at room temperature. Culture growth in this experiment mirrored that shown in Figure 4A such that the growth defect of the ElyC^−^ cells was not apparent until approximately 8 hours post-inoculation, or just over one doubling of the culture following the initial signs of a PG synthesis defect. The observation that growth can continue without PG synthesis for about one mass doubling is consistent with prior results of Prats and de Pedro [44] demonstrating that *E. coli* cells can grow normally with up to 50% less PG per cell.

Also consistent with a PG synthesis defect in the ElyC^−^ mutant, when PG sacculi were purified from unlabeled room temperature cultures of a Δ*elyC* strain harvested prior to observable cell lysis, the PG pellet obtained following boiling in 4% SDS was much smaller than the corresponding pellet from wild-type cells (Figure 5C). Pellet size was restored in Δ*elyC* cells expressing *elyC* in *trans* (Figure 5C). We conclude that ElyC^−^ cells have a severe defect in PG biogenesis at low temperatures.

### Multicopy suppression of the PG biogenesis defect in ElyC^−^ cells

**Figure 6:**
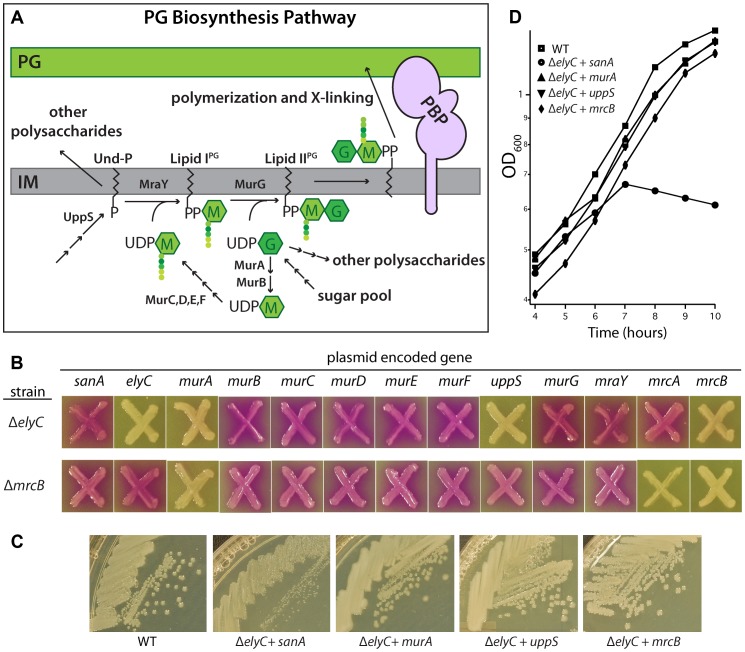
Suppression of the ElyC^−^ phenotypes. A. Diagram of the PG synthesis pathway. See text for details. M, MurNAc; G, GlcNAc. Colored circles represent amino acids in the PG stem peptide. B. Cells of EM1 [Δ*elyC*] and CB3 [Δ*mrcB*] containing multicopy plasmids with the indicated genes were patched onto CPRG indicator agar and grown as described for Figure 3B. Plasmids were selected from an ordered ORF library set [47]. C. A subset of the strains from B was grown on LB (1% NaCl) agar at room temperature and plates were photographed. D. The indicated subset of these strains was also grown in liquid and monitored as described for Figure 4A except that the medium was supplemented with 100 µM IPTG. In B–D, the WT strain did not contain any plasmid. Antibiotics were not included in the plates or in the liquid medium to select for the plasmid.

The PG synthesis pathway takes place in three stages (Figure 6A) [39]. Precursor biogenesis begins in the cytoplasm with the conversion of UDP-N-acetylglucosamine (UDP-GlcNAc) to UDP-N-acetylmuramic acid (UDP-MurNAc) by MurA and MurB. The peptide moiety is then added to UDP-MurNAc by MurC, D, E, and F, ultimately forming UDP-MurNAc-pentapeptide (UDP-MurNAc-pep). In the second, membrane-associated phase, UDP-MurNAc-pep is converted to the precursor lipid-I^PG^ by MraY, which transfers phospho-MurNAc-pep to the lipid carrier undecaprenol-phosphate (Und-P). Und-P is synthesized by the enzyme UppS. Lipid-II^PG^ is formed by MurG via the addition of GlcNAc to lipid-I^PG^ from UDP-GlcNAc. This final precursor contains the basic monomeric unit of PG, the disaccharide-pentapeptide. Following its production, lipid-II^PG^ must be flipped to expose the sugar units to the periplasmic space where it can then be polymerized and crosslinked into PG by the PBPs. The identity of the flippase remains controversial [45], [46].

To determine what stage of PG biogenesis might be affected by ElyC inactivation, we took a candidate approach to identify multi-copy suppressors of the ElyC growth and lysis phenotypes. Clones from a multi-copy plasmid ORF library [47] encoding untagged enzymes in the PG synthesis pathway expressed from a ColE1 plasmid under control of the *tac* promoter were selected and introduced into Δ*elyC* cells. The CPRG phenotype of the resulting strains was then assessed (Figure 6B). As expected, a plasmid harboring *elyC* suppressed the CPRG+ phenotype of a Δ*elyC* mutant. This was not the case for plasmids encoding *sanA*, the other DUF218 factors, or most of the enzymes involved in PG precursor biogenesis (Figure 6B–C, data not shown). Strikingly, however, overproduction of MurA, UppS, or PBP1b fully suppressed the CPRG+ phenotype of ElyC^−^ cells and restored their growth at room temperature to normal (Figure 6B–D). Interestingly, each of the enzymes with suppression activity functions at a major transition point in PG biogenesis. MurA catalyzes the committed step for PG synthesis [48], UppS is responsible for producing the lipid carrier Und-P, which is likely limiting for the synthesis of lipid-linked precursors [49], and PBP1b performs the final polymerization and crosslinking reactions [40]. Thus, overproduction of these factors may generally increase the flux through the pathway to alleviate the ElyC^−^ defect. To determine the specificity of the observed suppression, we monitored the ability of the plasmid set to correct the CPRG+ phenotype of a mutant lacking the PG synthase PBP1b (Figure 6B). The *murA*-containing plasmid retained suppressing activity in this background as did the PBP1b-encoding plasmid as expected. Interestingly, overproduction of PBP1a suppressed the CPRG+ phenotype of PBP1b^−^ cells but not that of the Δ*elyC* mutant (Figure 6B), suggesting that PBP activity is not generally limiting in cells lacking ElyC. On the other hand, UppS overproduction appeared to specifically suppress the loss of ElyC function (Figure 6B–D). We therefore infer that the primary defect in ElyC^−^ cells is likely to be at the level of lipid carrier metabolism.

### Genetic interactions between ElyC and the enterobacterial common antigen biogenesis pathway

Further support for a functional role for ElyC in lipid carrier metabolism was uncovered using high-throughput genetic interaction analysis technology (GIANT-coli) [50]. An Hfr donor strain harboring a Δ*elyC*::Cam^R^ allele was crossed with the Keio collection *en masse* to search for deletion alleles that either suppress the growth defect resulting from the loss of ElyC function at room temperature or exacerbate the Δ*elyC* phenotype at higher temperatures (30 and 37°C). Interestingly, the analysis identified both positive and negative interactions between Δ*elyC* and deletions of genes coding for enzymes involved in the biogenesis of enterobacterial common antigen (ECA), a surface polysaccharide produced by all enteric bacteria [51] (Table 1). The ECA polysaccharide is assembled from repeating units of GlcNAc, N-acetyl-D-mannosaminuronic acid (ManNAcA), and 4-acitamido-4,6-dideoxy-D-galactose (Fuc4NAc). It is commonly found as either a cyclic form in the periplasm or linked to phosphatidylglycerol in the outer membrane, but it can also be found attached to lipid A-core in some bacteria [51], [52]. Despite its ubiquity in enterobacteria, the exact function of ECA remains unclear. However, the polymer has been implicated in acid and bile-salt resistance and has been shown to be important for virulence [53]–[55].

**Table 1:** Genetic interactions between *elyC* and enterobacterial common antigen biosynthesis genes revealed by high-throughput GIANT coli analysis.

Suppressive interactions at room temperature	
Gene Name	Growth Score^a^
*wecG*	1.93
*wecC*	1.65
*wecA*	1.40

Negative interactions at 30°C	
Gene Name	Growth Score^a^
*wecE*	0.17
*wecF*	0.24
*rmlA^ECA^*	0.27

^a^ Growth score reflects the colony size of the double mutant clone in question divided by the average colony size of library clones. Scores greater than one indicate suppressive interactions and those less than one indicate negative interactions.

**Figure 7:**
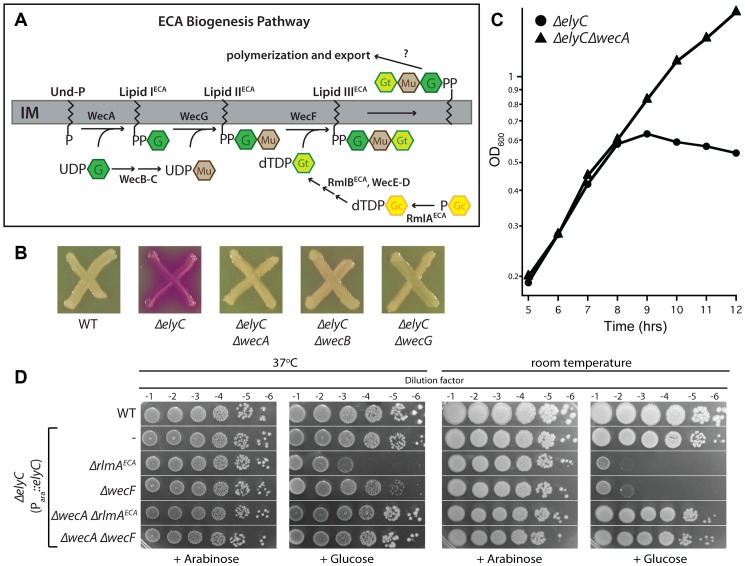
Genetic interaction between ElyC and the ECA biogenesis pathway. A. Diagram of the ECA precursor synthesis pathway. G, GlcNAc; Mu, ManNAcA; Gt, Fuc4NAc; P-Gc, glucose-1-phosphate. B. Cells of MG1655 [WT], EM9 [Δ*elyC*], CB329 [Δ*elyC* Δ*wecA*], CB337 [Δ*elyC* Δ*wecB*], and CB265 [Δ*elyC* Δ*wecG*] were patched onto CPRG indicator agar and grown as described for Figure 3B. C. The indicated subset of these strains was also grown in liquid LB 1% NaCl at room temperature and monitored as described for Figure 4A. Cultures of Δ*elyC* Δ*wecB* and Δ*elyC* Δ*wecG* strains grew as well as the Δ*elyC* Δ*wecA* strain (data not shown). D. Overnight cultures of TB28 [WT] or CB152(attλCB118) [Δ*elyC* (P_ara_::*elyC*)] and its Δ*rlmA* ^ECA^, Δ*wecF*, Δ*wecA* Δ*rlmA* ^ECA^, and Δ*wecA* Δ*wecF* derivatives were grown in LB supplemented with 0.2% arabinose and serially diluted following normalization for culture OD_600_. Five microliters of each dilution was spotted onto LB 1% NaCl agar supplemented with 0.2% arabinose or glucose as indicated and plates were incubated at the indicated temperature.

The steps required for the synthesis of the ECA precursor, lipid-III^ECA^, have been elucidated (Figure 7A) [56]–[58]. Like PG, its production proceeds via the progressive addition of sugar units to the lipid carrier Und-P. The genetic interaction analysis indicated that blocking lipid-I^ECA^ synthesis or its utilization suppressed the growth defect of Δ*elyC* cells grown at room temperature (Table 1). To confirm this observation, we transduced the Δ*wecA*::Kan^R^, Δ*wecB*::Kan^R^, and Δ*wecG*::Kan^R^ alleles from the Keio collection into a Δ*elyC* strain (MG1655 Δ*elyC::frt*) and assessed the phenotypes of the resulting transductants. Strikingly, all three Δ*wec* alleles fully suppressed the CPRG+ phenotype and growth defect of ElyC^−^ cells on solid medium, and the Δ*wecA*::Kan^R^ allele was shown to completely rescue the growth defect of Δ*elyC* cells in liquid medium at room temperature (Figure 7B–C). As opposed to the positive effects of blocks early in the ECA pathway, the genetic interaction analysis suggested that defects in lipid-II^ECA^ utilization adversely impact the growth of Δ*elyC* mutants. We tested this observation by transducing the Δ*rmlA^ECA^*::Kan^R^ and Δ*wecF*::Kan^R^ alleles from the Keio collection into an ElyC depletion strain CB152(attλCB118) [Δ*elyC* (P_ara_::*elyC*)]. The resulting transductants failed to grow at room temperature in the absence of *elyC* induction with arabinose (Figure 7D), indicating that the simultaneous inactivation of RmlA^ECA^ or WecF and ElyC is lethal under these conditions. Interestingly, depletion of ElyC in the Δ*rmlA^ECA^* or Δ*wecF* backgrounds also resulted in a plating defect at 37°C (Figure 7D), suggesting that ElyC function is not limited to low temperatures. The negative interaction between Δ*elyC* and Δ*rmlA^ECA^* or Δ*wecF* suggests that the accumulation of the intermediate precursor lipid-II^ECA^ is toxic when ElyC is inactivated. Accordingly, inactivation of WecA suppressed the observed synthetic lethal phenotype displayed by ElyC^−^ RmlA^ECA−^ and ElyC^−^ WecF^−^ cells (Figure 7D).

The genetic interactions between *elyC* and the ECA biosynthesis genes are consistent with the observed suppression of the ElyC^−^ phenotype by the overproduction of the Und-P synthase UppS. Taken together, these findings suggest that in the absence of ElyC, the PG biogenesis pathway is hypersensitive to competition for the lipid carrier from ECA synthesis and potentially the synthesis of other surface polysaccharides that utilize Und-P. In this context, it is the basal level of competition for Und-P due to ECA synthesis that likely results in the baseline growth defect of ElyC^−^ cells. We suspect that this competition is intensified when flux through the ECA pathway is blocked at the stage of lipid-II^ECA^ utilization, resulting in the observed synthetic lethal phenotypes due to the build-up of this precursor and the further depletion of the free Und-P pool. Mutations that lead to a defect in lipid-I^ECA^ utilization are likely to be suppressive rather than negative because the WecA reaction is readily reversible. Overall, our genetic analysis of ElyC function is consistent with a model in which it plays an important role in Und-P metabolism, possibly by promoting the efficient utilization of Und-P or Und-P-linked precursors by the PG biogenesis pathway. The example of ElyC thus clearly demonstrates the utility of the high-throughput CPRG screening method in uncovering new factors involved in important aspects of envelope biogenesis.

## Discussion

The cell envelope of Gram-negative bacteria is a formidable barrier that is difficult for antimicrobial drugs to penetrate. Thus, the list of treatments effective against these organisms is small and shrinking rapidly with the rise of new resistance mechanisms, especially those possessed by carbapenem-resistant *Enterobacteriaceae* [59]. To address this problem, input from all levels of the scientific endeavor is required, from the most fundamental to the applied. Key to success in combating resistance will be a detailed mechanistic understanding of the cell envelope assembly process. Great strides have been made towards this goal, particularly in recent years with the identification of the essential machineries required for outer membrane biogenesis [7]. However, many aspects of Gram-negative envelope assembly remain poorly characterized, including the regulatory strategies used to coordinate the construction of the different envelope layers. To shed light on these and other aspects of envelope assembly, we developed the high-throughput screening platform described in this report. A number of factors with previously described roles in envelope assembly were positively identified in our screen, indicating that it works as intended. Moreover, the screen also implicated many genes of currently unknown function as being important for envelope integrity. Further study of these factors is likely to reveal new mechanisms underlying the envelope assembly process, any one of which could serve as a drug target to either block cell growth or render the envelope permeable to approved drugs currently ineffective against Gram-negative organisms.

Our discovery and characterization of ElyC as a new envelope biogenesis factor validates the utility of the CPRG screening approach. ElyC belongs to a broadly conserved family of proteins with the DUF218 domain. Even though a wide range of bacteria encode factors with DUF218 domains [19], the function(s) of these proteins have remained largely mysterious. At higher temperatures, mutants lacking ElyC grow normally. However, when grown at room temperature, ElyC inactivation results in a striking lysis phenotype. Our genetic and physiological studies indicate that cell lysis at low temperatures is due to a severe defect in PG synthesis. Because this phenotype can be overcome by the overproduction of the lipid carrier synthase UppS, we infer that mutants lacking ElyC are impaired in the lipid stages of the pathway. Consistent with this observation, mutations that disrupt the synthesis of the ECA polysaccharide and lead to the accumulation of lipid intermediates in its synthesis were found to be synthetically lethal with an ElyC defect. This phenotype was caused by the accumulation of ECA lipid intermediates and not the loss of ECA production because inactivating the first enzyme in the pathway, WecA, suppressed the synthetic lethality. Surprisingly, we found that a WecA defect also suppressed the baseline CPRG+ phenotype and growth defect of a mutant lacking ElyC alone. This observation suggests that the phenotypes displayed by a Δ*elyC* mutant are likely to be the result of competition between the PG and ECA synthetic pathways for the lipid carrier Und-P. Competition is likely heightened when the ECA pathway is impaired and its lipid intermediates accumulate [55], thus causing a greater drain on the Und-P pool and the observed synthetic lethal phenotypes.

The connection between ElyC and ECA synthesis provides a likely explanation for the temperature-dependent nature of the phenotypes displayed by a Δ*elyC* mutant. In the related bacterium *Yersinia enterocolitica*, it was recently shown that ECA production is higher at 22°C relative to 37°C, and that this correlates with an increase in the expression of the ECA biosynthetic cluster at low temperature [60]. Thus, we suspect that increased ECA synthesis at low temperatures is likely to place additional demands on the lipid carrier pool that reveals the defect in Und-P metabolism resulting from the loss of ElyC function. ECA biogenesis may also increase as cells enter late-exponential phase at room temperature, thus providing a potential explanation for why Δ*elyC* cells grow normally until this stage of growth. Interestingly, the observed negative genetic interaction between Δ*elyC* and either Δ*rmlA^ECA^* or Δ*wecF* at 37°C suggests that ElyC function is not restricted to low temperatures, but rather its function just becomes more important as the temperature drops.

Although the technical challenges of working with low abundance phospholipids has thus far prevented us from pinpointing the exact function of ElyC, our genetic analysis suggests a potential role for ElyC in lipid-II^PG^ metabolism. Overproduction of MurA and UppS are both likely to suppress the ElyC defect by enhancing lipid-II^PG^ synthesis. The former likely does so by increasing the flux through the PG synthesis pathway and the latter by increasing the pool size of Und-P available for the lipid-precursor generating enzymes. We therefore infer that cells defective for ElyC may either have reduced lipid-II^PG^ levels and/or may not efficiently utilize the lipid-II^PG^ that is produced. According to this view, overproduction of PBP1b is probably able to suppress the ElyC^−^ defect by providing more synthases to overcome a potentially lower effective lipid-II^PG^ concentration. If this interpretation is correct, the PG synthesis defect of ElyC^−^ cells may be most apparent when the synthesis of other polysaccharides is induced because ElyC functions to preferentially “funnel” lipid carrier or precursors through the PG pathway. One attractive possibility is that ElyC is part of a mechanism that enhances the affinity of MraY for Und-P so that PG synthesis is the dominant pathway for lipid carrier utilization. Alternatively, ElyC may help the PBPs properly select lipid-II^PG^ over the many other lipid-linked precursors likely to be present in the cell membrane at times when other extracellular polysaccharides are being produced at high levels. Other functions for ElyC in lipid carrier metabolism are also consistent with the data, including a role in Und-P production or its recycling, and indirect effects are difficult to exclude at this stage. Nevertheless, given the central location of Und-P in the synthetic pathways of all manner of extracellular polysaccharides, any of the aforementioned roles for ElyC has important implications for our understanding of envelope biogenesis. Further study of its function and that of other factors identified in our screen is therefore likely to reveal new and interesting ways to disrupt the envelope assembly process for therapeutic purposes.

An exciting aspect of the CPRG assay is the potential for expanding its use in larger scale chemical genomics analyses. Using only four conditions in the current study, we were able to identify phenotypes for ∼80 mutants of genes of unknown function that were previously unresponsive when using colony size alone as a proxy of fitness in >300 conditions [31]. Although there are aspects of the assay that still need to be optimized before it can be expanded to a large number of growth conditions (color diffusion and timing of color development), its use as a highly sensitive readout for chemical genomics studies holds great promise for uncovering gene function and pathway organization based on the similarity of the response profiles for different mutants. Furthermore, because of its simplicity and use of the widely employed LacZ reporter, the CPRG assay described here should be readily adaptable to other organisms and enable similar high-throughput screens to discover envelope biogenesis factors in bacteria with distinct surface architectures.

## Materials and Methods

### Media, bacterial strains, and plasmids

Cells were grown in LB [1% tryptone, 0.5% yeast extract, and 0.5% NaCl], unless the salt concentration is specified otherwise (0 or 1% NaCl). Antibiotics were used at 10 (chloramphenicol; Cm), 15 (ampicillin; Amp) or 20 (kanamycin; Kan) µg/ml. Bacterial strains used are listed in Table S5. All *E. coli* strains used in the reported experiments are derivatives of MG1655. Plasmids used in this study are listed in Table S6. Plasmids from the multicopy ORF library [47] used throughout this study encode untagged proteins expressed from a ColE1-derived vector under control of the *tac* promoter. See Text S1 for plasmid construction details.

### Screening of transposon library for envelope defective mutants

Wild-type MG1655 cells were mutagenized with the EzTn-Kan transposome from Epicentre as described previously [28]. Cells from the resulting library were plated on LB (0.5% NaCl) agar supplemented with CPRG (20 µg/ml) and IPTG (50 µM) to achieve a density of about 100 colonies per plate. After approximately 15–28 hours of growth at 30°C, colonies displaying a red or strong pink CPRG+ phenotype were purified on LB agar prepared with 0, 0.5, and 1% salt and grown at 30°C and 42°C overnight. Cells from colonies of mutants grown under each condition were visualized by phase contrast microscopy. The location of the transposon insertions in about 100 CPRG+ mutants was determined using arbitrarily primed PCR and DNA sequencing as described previously [28].

### Screening of the ordered library for CPRG+ mutants

A copy of the previously described ordered *E. coli* mutant library [31] stored in 96-well format at −80°C was thawed, pinned onto LB-Kan agar plates, and grown overnight at 37°C. The following day the library was condensed to 384-pin format on LB-Kan agar plates using a Singer Rotor robot. After growth overnight, the library was transferred to LB agar plates spread with 100 µl of an overnight culture of JA200/pCB112 [donor strain/P_lac_::*lacZ*, Cam^R^]. The resulting mating plates were incubated overnight at 37°C, positions corresponding to the ordered library were transferred to LB-Kan-Cam plates, and the plates were incubated again at 37°C overnight. Finally, the Lac+ exconjugants of the ordered library were transferred to LB agar supplemented with CPRG (20 µg/ml), IPTG (100 µM), and various NaCl concentrations (0, and 1%), and incubated either at room temperature or at 30°C. Plates were monitored through time and were imaged both at the end of vegetative growth (∼12 or 23 hours for the 30°C or room temperature grown cells, respectively), and after their growth plateaued, 7 hours later. Images were analyzed using in-house software (Iris) that segments the image and measures the hue of each pixel in the HSV color space. The score for each mutant is an average of the scores of the 2 clones present in the library for each mutant. CPRG scores for the early time-points were used for the subsequent analysis. Thresholds were set as described in the text and were manually evaluated to minimize false negatives and false positives.

### Assessment of phenotypes and multicopy suppression analysis

To monitor the growth of Δ*elyC* cells in liquid medium, overnight cultures of TB28 [WT] and CB152 [Δ*elyC*] strains were grown in LB at 37°C. These cultures were diluted 1∶100 in LB 1% NaCl and grown at 37°C to an OD_600_ of approximately 0.4. Cultures were then diluted to an OD_600_ of 0.04 in LB and grown at room temperature in a shaking water bath. Measurements of the culture OD_600_ were then taken every 30–60 minutes.

For viability measurements, overnight cultures were adjusted to an OD_600_ of 2, serial dilutions from 10^−1^ to 10^−6^ were prepared in LB, and 5 µL of each dilution were spotted onto solid medium. Plates were incubated overnight at 37°C or 3 days at room temperature and photographed. To assess the CPRG phenotypes of various strains, single colonies from a freshly streaked LB agar plate were patched in the shape of an X onto LB medium containing 20 µg/ml of CPRG and 50 µM of IPTG. Plates were incubated overnight at room temperature and photographed. For strains containing multicopy plasmids, the CPRG phenotypes were assessed identically except that the concentration of IPTG was increased to 100 µM.

### Measurement of PG biogenesis and sacculi preparation

Overnight cultures of CB74 [*lysA*::Tn*10*] and CB330 [Δ*elyC lysA*::Tn*10*] were grown in LB medium supplemented with 100 µg/ml lysine. A *lysA*::Tn*10* strain background was used to prevent the incorporation of added mDAP into proteins. The overnight cultures were diluted 1∶100 and grown in LB to an OD_600_ of about 0.4 as above. The cultures were then diluted to an OD_600_ of 0.04 into LB 1% NaCl supplemented with lysine, methionine and threonine at 100 µg/ml and [^3^H]-mDAP (American Radiolabeled Chemicals) at 10 µCi/ml. Cultures were grown at room temperature in a shaking water bath and at the time points indicated in Figure 5B, 0.2 ml of culture was withdrawn and added to 0.8 ml of hot (95°C) 5% SDS solution. Samples were incubated at 95°C for an additional 30 minutes, cooled to room temperature, and SDS-insoluble PG material was recovered by filtration essentially as described [61]. Filters were dried and radioactivity detected by scintillation counting using a Microbeta Trilux 1450 LSC from Perkin-Elmer and Ecolite (MP biomedicals) scintillation fluid.

Sacculi pellets were prepared for TB28 [WT], CB152 [Δ*elyC*], and CB152(attλCB118) [Δ*elyC* (P_ara_::*elyC*)]. Overnight cultures were diluted and grown to exponential phase as described above. They were then diluted into 500 ml of LB 1% NaCl and grown at 37°C or room temperature to an OD_600_ of 0.5. Cultures of CB152(attλCB118) additionally contained 0.2% arabinose. Cells were harvested by centrifugation at 4000× g for 10 minutes at 4°C and the pellet was resuspended in 10 ml of ice-cold phosphate buffered saline. The cell suspension was added drop-wise to 40 ml of boiling SDS 5% solution. The mixtures were boiled for 30 minutes with stirring, and left to cool at room temperature overnight. Sacculi were sedimented by ultracentrifugation at 100,000 g for 1 h at 25°C and the resulting cell pellets were photographed.

### High-throughput genetic interaction analysis

The ordered library described above was further condensed to 1536-pin format and transferred to plates spread with 50 µl of a culture of CB157 [Hfr donor, Δ*elyC*::*cat*] at an OD_600_ of 1. The mating plates were incubated overnight and after an intermediate selection against the donor parent, double mutants were selected in LB plates containing both antibiotics [50]. Interactions were scored by directly comparing the growth of the double mutants to that of the KEIO single mutants (Table 1).

## Supporting Information

Figure S1 Score distributions for the CPRG analysis. A–C. CPRG score distributions for the screen carried out under the indicated conditions. Positions of genes of interest and/or known importance for envelope integrity are indicated. Genes with scores above the cut-off (10^3.7^ units) were designated as CPRG+ hits.
(TIF)

Table S1 Scores from CPRG analysis of the mutant collection. Five tabs in the spreadsheet are included. 30 LB0, 30 LB1, RT LB0, RT LB1, and gene names for mutants above threshold. The first four tabs provide average CPRG scores from the analysis at 30°C LB no salt, 30°C LB 1% NaCl, room temperature LB no salt, and room temperature LB 1% salt. The final tab lists gene names for those mutants scoring above the threshold of 10^3.7^ arbitrary units.
(XLSX)

Table S2 Gene Ontology (GO) enrichment. The spreadsheet lists terms and p-values for their relative enrichments among the hits identified in the CPRG screen.
(XLSX)

Table S3 KEGG pathway enrichment. The spreadsheet lists pathways and p-values for their relative enrichments among the hits identified in the CPRG screen.
(XLSX)

Table S4 Orphan hits identified in the CPRG screen. The file lists the gene names of all genes of unknown function (orphans) identified as possible envelope assembly factors in the CPRG screen.
(XLSX)

Table S5 Lists strains used in this study.
(DOC)

Table S6 Lists plasmids used in this study.
(DOC)

Text S1 Supplemental methods and materials. Details for plasmid constructions and other supplementary protocols are given.
(DOC)
