# Supplementary figures and images for: A Genome-Wide Screen for Bacterial Envelope Biogenesis Mutants Identifies a Novel Factor Involved in Cell Wall Precursor Metabolism
Source: PLoS Genet. 2014 Jan 2;10(1):e1004056. doi: 10.1371/journal.pgen.1004056 (PMC3879167; doi:10.1371/journal.pgen.1004056)

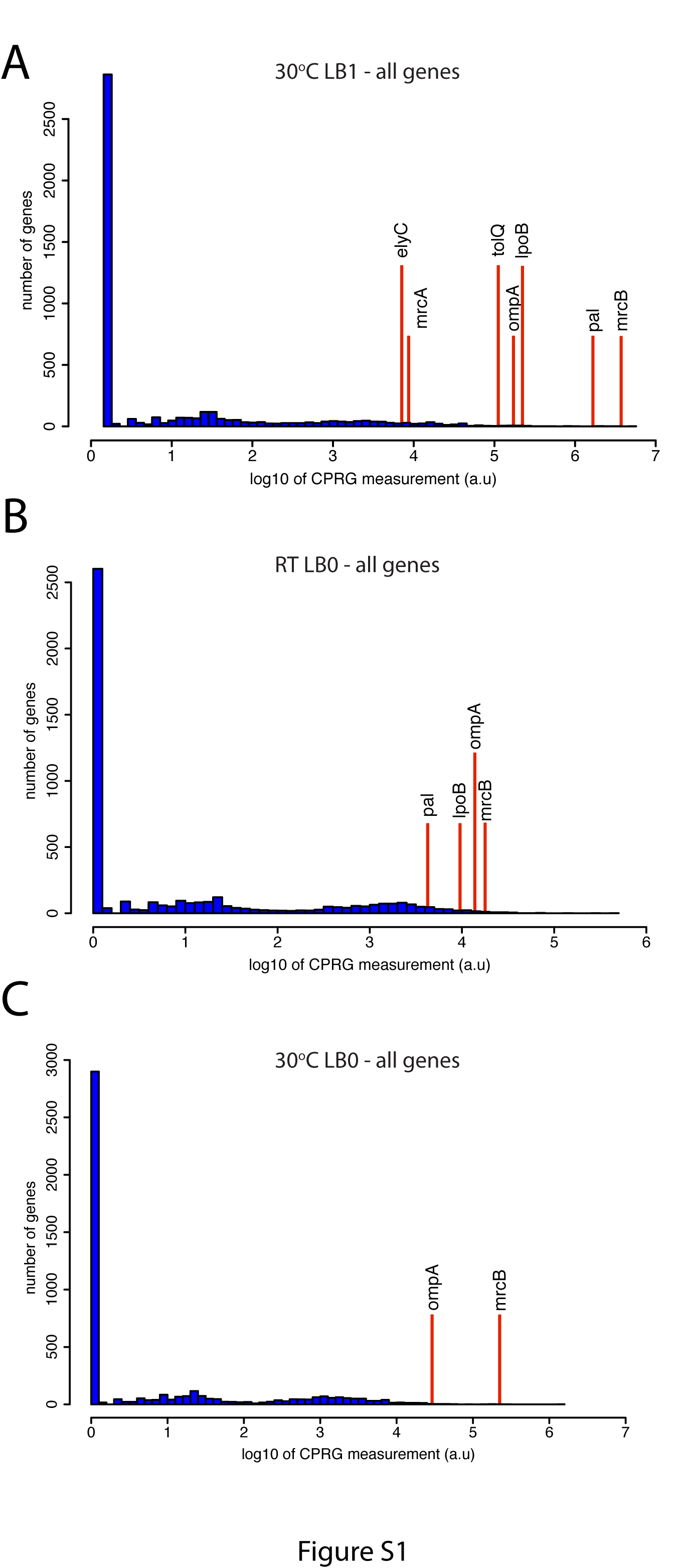

Supplement: Figure S1 — Score distributions for the CPRG analysis. A–C. CPRG score distributions for the screen carried out under the indicated conditions. Positions of genes of interest and/or known importance for envelope integrity are indicated. Genes with scores above the cut-off (103.7 units) were designated as CPRG+ hits. (TIF) [file pgen.1004056.s001.tif]
