# Supplementary material for: A Genome-Wide Screen for Bacterial Envelope Biogenesis Mutants Identifies a Novel Factor Involved in Cell Wall Precursor Metabolism
Source: PLoS Genet. 2014 Jan 2;10(1):e1004056. doi: 10.1371/journal.pgen.1004056 (PMC3879167; doi:10.1371/journal.pgen.1004056)
Supplement: Table S5 — Lists strains used in this study. (DOC) [file pgen.1004056.s006.doc]

**Table S5**. Strains used in this study.

| Strain | Genotype^a^ | Source/Reference^b^ |
| --- | --- | --- |
| DH5α | F– hsdR17 deoR recA1 endA1 phoA supE44 thi-1 gyrA96 relA1 Δ(lacZYA-argF)U169 ϕ80dlacZΔM15 | Gibco BRL |
| MG1655 | rph1 ilvG rfb-50 | [4] |
| JA200 | F+, thr-1, leu-6, DE(trpE)5, recA, lacY, thi, gal, xyl, ara, mtl | [2] |
| BW25113 | Δ(araD-araB)567 ΔlacZ4787(::rrnB-3) rph-1 Δ(rhaD-rhaB)568 hsdR514 | [2] |
| JW0903-1 | BW35113 ΔelyC::Kan^R^ | [2] |
| JW0145-1 | BW35113 ΔmrcB::Kan^R^ | [2] |
| JW3359-1 | BW35113 ΔmrcA::Kan^R^ | [2] |
| JW1411-1 | BW35113 ΔydcF::Kan^R^ | [2] |
| JW2132-1 | BW35113 ΔsanA::Kan^R^ | [2] |
| JW3057-1 | BW35113 ΔygjQ::Kan^R^ | [2] |
| JW3758-2 | BW35113 ΔwecA::Kan^R^ | [2] |
| JW5600-1 | BW35113 ΔwecB::Kan^R^ | [2] |
| JW3770-1 | BW35113 ΔwecG::Kan^R^ | [2] |
| JW5596-1 | BW35113 ΔwecF::Kan^R^ | [2] |
| JW3763-2 | BW35113 ΔrmlA^ECA^::Kan^R^ | [2] |
| TB28 | MG1655 ΔlacIZYA::frt | [3] |
| EM1 | MG1655 ΔelyC::Kan^R^ | P1(JW0903-1) X MG1655 |
| EM2 | MG1655 ΔydcF::Kan^R^ | P1(JW1411-1) X MG1655 |
| EM3 | MG1655 ΔsanA::Kan^R^ | P1(JW2132-1) X MG1655 |
| EM4 | MG1655 ΔygjQ::Kan^R^ | P1(JW3057-1) X MG1655 |
| EM8 | MG1655 ΔygjQ::frt sanA::kan | P1(JW2132-1) X EM12 |
| EM9 | MG1655 ΔelyC::frt | EM1/pCP20 |
| EM12 | MG1655 ΔygjQ::frt | EM4/pCP20 |
| EM13 | MG1655 ΔygjQ::frt ΔsanA::frt | EM12/pCP20 |
| EM14 | MG1655 ΔygjQ::frt ΔsanA::frt ΔydcF::Kan^R^ | P1(JW1411-1) X EM13 |
| EM15 | MG1655 ΔygjQ::frt ΔsanA::frt ΔydcF::frt | EM14/pCP20 |
| EM16 | MG1655 (ΔygjQ::frt ΔsanA::frt ΔydcF::frt ΔelyC::Kan^R^ | P1(JW0903-1)X EM15 |
| TU73 | rph1 ilvG rfb-50 ampD::frt lysA::Tn10 | [7] |
| TU121 | TB28 mrcA::frt | [6] |
| TU122 | TB28 mrcB::frt | [6] |
| CAG61029 | Δ(araD-araB)567 ΔlacZ4787(::rrnB-3) rph-1 Δ(rhaD-rhaB)568 hsdR514 pseudoHfr::(trp gen (-)) | [5] |
| elyC::cat | BW25113 ΔelyC::Kan^R^ | [5] |
| CB151 | TB28 ΔelyC::Kan^R^ | P1(JW0903-1) X TB28 |
| CB152 | TB28 ΔelyC::frt | CB151/pCP20 |
| CB2 | MG1655 ΔmrcB::Kan^R^ | P1(JW0145-1) X MG1655 |
| CB171 | TB28 ΔelyC::frt ΔmrcA::Kan^R^ | P1(JW3359-1) X CB152 |
| CB172 | TB28 ΔelyC::frt ΔmrcB::Kan^R^ | P1(JW0145-1) X CB152 |
| CB74 | TB28 lysA::Tn10 | P1(TU73) X TB28 |
| CB330 | TB28 ΔelyC::frt lysA::Tn10 | P1(TU73) X CB152 |
| CB157 | Δ(araD-araB)567 ΔlacZ4787(::rrnB-3) rph-1 Δ(rhaD-rhaB)568 hsdR514 pseudoHfr::(trp gen (-)) elyC::cat | P1(elyC::cat) X CAG61029 |
| CB329 | MG1655 ΔelyC::frt ΔwecA::Kan^R^ | P1(JW3758-2) X CB235 |
| CB380 | MG1655 ΔelyC::frt ΔwecA::frt | CB329/pCP20 |
| CB337 | MG1655 ΔelyC::frt ΔwecB::Kan^R^ | P1(JW5600-1) X CB235 |
| CB265 | MG1655 ΔelyC::frt ΔwecG::Kan^R^ | P1(JW3770-1) X CB235 |
| CB335 | MG1655 ΔelyC::frt ΔwecF::Kan^R^ (P_ara_::elyC) | P1(JW5596-1) X CB152(attλCB118) |
| CB218 | MG1655 ΔelyC::frt ΔrmlA^ECA^::Kan^R^ (P_ara_::elyC) | P1(JW3763-2) X CB152(attλCB118) |
| CB381 | MG1655 ΔelyC::frt ΔwecA::frt ΔwecF::Kan^R^ | P1(JW5596-1) X CB380 |
| CB409 | MG1655 ΔelyC::frt ΔwecA::frt (P_ara_::elyC) | P1[CB152(attλCB118)] X CB380 |
| CB410 | MG1655 ΔelyC::frt ΔwecA::frt ΔrmlA^ECA^::Kan^R^ (P_ara_::elyC) | P1(CB218) X CB409 |
| CB411 | MG1655 ΔelyC::frt ΔwecA::frt ΔwecF::Kan^R^ (P_ara_::elyC) | P1(CB335) X CB409 |
| Ely7 | MG1655 nhaA::Tn5 | Transposon mutagenesis of MG1655 |

^a^ The Kan^R^ cassette is flanked by *frt* sites for removal by FLP recombinase. An *frt* scar remains following removal of the cassette using FLP expressed from pCP20.

^b^ Strain constructions by P1 transduction are described using the shorthand: P1(donor) x recipient. In all cases transductants were selected on LB Kan plates.
