# Supplementary material for: A Genome-Wide Screen for Bacterial Envelope Biogenesis Mutants Identifies a Novel Factor Involved in Cell Wall Precursor Metabolism
Source: PLoS Genet. 2014 Jan 2;10(1):e1004056. doi: 10.1371/journal.pgen.1004056 (PMC3879167; doi:10.1371/journal.pgen.1004056)
Supplement: Table S6 — Lists plasmids used in this study. (DOC) [file pgen.1004056.s007.doc]

**Table S6**. Plasmids used in this study.

| **Plasmid** | **Genotype** | **Origin** | **Source or reference** |
| --- | --- | --- | --- |
| pCB112 | cat lacIq P_lac_ ::lacZ | pBR | This work |
| pCB118 | attλ cat P_ara_ ::elyC | R6K | This work |
| ORF plasmid encoding elyC | bla lacIq P_tac_ ::elyC | pBR | [1] |
| ORF plasmid encoding sanA | bla lacIq P_tac_ ::sanA | pBR | [1] |
| ORF plasmid encoding ygjQ | bla lacIq P_tac_ ::ygjQ | pBR | [1] |
| ORF plasmid encoding ydcF | bla lacIq P_tac_ ::ydcF | pBR | [1] |
| ORF plasmid encoding murA | bla lacIq P_tac_ ::murA | pBR | [1] |
| ORF plasmid encoding murB | bla lacIq P_tac_::murB | pBR | [1] |
| ORF plasmid encoding murC | bla lacIq P_tac_ ::murC | pBR | [1] |
| ORF plasmid encoding murD | bla lacIq P_tac_ ::murD | pBR | [1] |
| ORF plasmid encoding murE | bla lacIq P_tac_ ::murE | pBR | [1] |
| ORF plasmid encoding murF | bla lacIq P_tac_ ::murF | pBR | [1] |
| ORF plasmid encoding uppS | bla lacIq P_tac_ ::uppS | pBR | [1] |
| ORF plasmid encoding murG | bla lacIq P_tac_ ::murG | pBR | [1] |
| ORF plasmid encoding mraY | bla lacIq P_tac_ ::muraY | pBR | [1] |
| ORF plasmid encoding mrcA | bla lacIq P_tac_ ::mrcA | pBR | [1] |
| ORF plasmid encoding mrcB | bla lacIq P_tac_ ::mrcB | pBR | [1] |

^a^ P_lac_, P_tac_, and P_ara_ indicate the lactose, tac, and arabinose promoters, respectively.
