# Supplementary material for: A Genome-Wide Screen for Bacterial Envelope Biogenesis Mutants Identifies a Novel Factor Involved in Cell Wall Precursor Metabolism
Source: PLoS Genet. 2014 Jan 2;10(1):e1004056. doi: 10.1371/journal.pgen.1004056 (PMC3879167; doi:10.1371/journal.pgen.1004056)
Supplement: Text S1 — Supplemental methods and materials. Details for plasmid constructions and other supplementary protocols are given. (DOC) [file pgen.1004056.s008.doc]

**A genome-wide screen for bacterial envelope biogenesis mutants identifies a novel factor involved in cell wall precursor metabolism**

Catherine Paradis-Bleau^1^, George Kritikos^3^, Katya Orlova^2^, Athanasios Typas^3^, and Thomas G. Bernhardt^4^*

^1^Department of Microbiology, Infectiology and Immunology

Université de Montréal

Montréal (Québec) H3C 3J7

^2^Department of Microbiology and Immunology

University of California at San Francisco

San Francisco, CA 94158-2517

^3^ European Molecular Biology Laboratory

Genome Biology Unit

Heidelberg 69117, Germany

^4^Department of Microbiology and Immunobiology

Harvard Medical School

Boston, MA 02115

*To whom correspondence should be addressed.

Thomas G. Bernhardt, Ph.D.

Harvard Medical School

Department of Microbiology and Immunobiology

Boston, Massachusetts 02115

e-mail: [thomas_bernhardt@hms.harvard.edu](mailto:thomas_bernhardt@hms.harvard.edu)

**Running title:** Rapid screen for envelope assembly factors

**Abbreviations:** CPRG, chlorophenyl red β-D-galactopyranoside;

**Keywords:** cell envelope/peptidoglycan/morphogenesis/cell wall/bacteriolysis

**Supplemental Methods and Materials**

**Plasmid construction**

Plasmids used in this study are listed below. In all cases PCR was performed using KOD polymerase (Novagen) according to the manufacturer’s instructions. Unless otherwise indicated, MG1655 chromosomal DNA was used as the template. Restriction sites for use in plasmid constructions are bold, italicized and underlined in the primer sequences given below. Plasmid DNA and PCR fragments were purified using the Qiaprep spin miniprep kit (Qiagen) or the Qiaquick PCR purification kit (Qiagen), respectively. Plasmids constructed by PCR were verified by DNA sequencing.

pCB112:

The lacZ gene was amplified using the forward primer 5’-GCTAGAATTCTGGTGGTCAGATGCGGGATG-3’ and the

reverse primer 5’GCTAAAGCTTCTGCTGAACGGCAAGCCGTTG-3’. The PCR product was inserted into several intermediate vectors, but ultimately used to construct a mobile plasmid based on that used for the ORF library [1].

pCB118:

The elyC gene was amplified using the primers 5’-GTCATCTAGAAAATAAGGAGATATACATATGCTTTTTACACTGAAAAAAGT -3’ and 5’ GTCAAAGCTTCGATTTAGTCGGCGCAACTTCGATC-3’. The PCR product and backbone plasmid pCB88 were digested with the enzymes XbaI and HindIII and the fragments were ligated to form pCB118. pCB88 contains a fusion of the bacteriophage MS2 L lysis gene fused to GFP under control of the arabinose promoter. The L-GFP fusion was replaced by *elyC* in the construction of pCB118. The construct is ultimately a derivative of pTB285 [8] and confers resistance to chloramphenicol and is a conditionally replicating vector that can be integrated at the bacteriophage lambda attachment site.

REFERENCES

1. Saka K, Tadenuma M, Nakade S, Tanaka N, Sugawara H, et al. (2005) A complete set of Escherichia coli open reading frames in mobile plasmids facilitating genetic studies. DNA Res 12: 63–68.

2. Baba T, Ara T, Hasegawa M, Takai Y, Okumura Y, et al. (2006) Construction of Escherichia coli K-12 in-frame, single-gene knockout mutants: the Keio collection. Mol Syst Biol 2: 2006.0008. doi:10.1038/msb4100050.

3. Bernhardt TG, de Boer PAJ (2004) Screening for synthetic lethal mutants in Escherichia coli and identification of EnvC (YibP) as a periplasmic septal ring factor with murein hydrolase activity. Molecular Microbiology 52: 1255–1269. doi:10.1111/j.1365-2958.2004.04063.x.

4. Guyer MS, Reed RR, Steitz JA, Low KB (1981) Identification of a sex-factor-affinity site in E. coli as gamma delta. Cold Spring Harb Symp Quant Biol 45 Pt 1: 135–140.

5. Typas A, Nichols R, Siegele D, Shales M, Collins S, et al. (2008) High-throughput, quantitative analyses of genetic interactions in E. coli. Nat Methods 5: 781-787. doi:10.1038/nmeth.1240.

6. Paradis-Bleau C, Markovski M, Uehara T, Lupoli TJ, Walker S, et al. (2010) Lipoprotein cofactors located in the outer membrane activate bacterial cell wall polymerases. Cell 143: 1110–1120. doi:10.1016/j.cell.2010.11.037.

7. Uehara T, Park JT (2008) Growth of Escherichia coli: significance of peptidoglycan degradation during elongation and septation. J Bacteriol 190: 3914–3922. doi:10.1128/JB.00207-08

8. Uehara T, Parzych KR, Dinh T, Bernhardt TG (2010) Daughter cell separation is controlled by cytokinetic ring-activated cell wall hydrolysis. EMBO J 29: 1412–1422. doi:10.1038/emboj.2010.36.
